# Jefferson Medical College

**Published:** 1892-06

**Authors:** 


### The Jefferson Medical College of Philadelphia.

The Board of Trustees and the Faculty of Jefferson Medical College are about to erect a handsome hospital, lecture hall, and laboratory building. The estimated cost of the building is $500,000. With the hospital on one side affording clinical facilities and the laboratory on the other side of the college hall for scientific research and training, the college will be most favourably situated for giving thorough instruction in medicine. The move has been made necessary by the large number of students who are now being instructed in this institution and because the Faculty desire to keep the school and hospital in the foremost rank of medical education in the United States.

